# Can developmental trajectories in gait variability provide prognostic clues in motor adaptation among children with mild cerebral palsy? A retrospective observational cohort study

**DOI:** 10.3389/fnhum.2023.1205969

**Published:** 2023-09-19

**Authors:** Rosa M. S. Visscher, Michelle Gwerder, Elke Viehweger, William R. Taylor, Reinald Brunner, Navrag B. Singh

**Affiliations:** ^1^Department of Health Science & Technology, Laboratory for Movement Biomechanics, Institute for Biomechanics, ETH Zürich, Zürich, Switzerland; ^2^Department of Biomedical Engineering, University of Basel, Basel, Switzerland; ^3^Laboratory of Movement Analysis, University Children's Hospital Basel (UKBB), Basel, Switzerland; ^4^Singapore-ETH Centre, Future Health Technologies Program, CREATE Campus, Singapore, Singapore

**Keywords:** gait analysis, developmental trajectories, gait variability, cerebral palsy, gross motor function classification scale (GMFCS)

## Abstract

**Aim:**

To investigate whether multiple domains of gait variability change during motor maturation and if this change over time could differentiate children with a typical development (TDC) from those with cerebral palsy (CwCP).

**Methods:**

This cross-sectional retrospective study included 42 TDC and 129 CwCP, of which 99 and 30 exhibited GMFCS level I and II, respectively. Participants underwent barefoot 3D gait analysis. Age and parameters of gait variability (coefficient of variation of stride-time, stride length, single limb support time, walking speed, and cadence; as well as meanSD for hip flexion, knee flexion, and ankle dorsiflexion) were used to fit linear models, where the slope of the models could differ between groups to test the hypotheses.

**Results:**

Motor-developmental trajectories of gait variability were able to distinguish between TDC and CwCP for all parameters, except the variability of joint angles. CwCP with GMFCS II also showed significantly higher levels of gait variability compared to those with GMFCS I, these levels were maintained across different ages.

**Interpretation:**

This study showed the potential of gait variability to identify and detect the motor characteristics of high functioning CwCP. In future, such trajectories could provide functional biomarkers for identifying children with mild movement related disorders and support the management of expectations.

## 1. Introduction

Walking is an essential activity of daily living (ADL) that supports independent living. Humans typically learn to walk in a bipedal manner early in life and can perform its elementary constituent – a step – around the age of 1 year. Step repetitions, including both stance and swing phases separated by touch-down and lift-off events, are integral for continuous walking. Here, seamless transition between phases and events requires propulsion for forward motion, and bodyweight to be shifted between limbs, all while coordinating foot placement to maintain balance and avoid falling. Continuous walking is therefore a complex undertaking that requires a multitude of motor skills to be learned, and achievement of developmental milestones (Thelen and Ulrich, 1991).

The process of learning how to walk is generally unstable, variable, and inefficient (Hallemans et al., 2006). However, during the first years of walking, children are generally able to optimize their movement patterns, and this learning process is underlined by multifaceted changes in their neurological control and motor systems (Thelen and Ulrich, 1991; Bartlett and Palisano, 2000). This observable and quantifiable adaptation of walking skills has led to a large body of literature, indicating that maturation of walking likely continues into adolescence (Hausdorff et al., 1985; Gouelle et al., 2016; Bisi et al., 2019; Kung et al., 2019).

The entire process of gait development is considered complex even in typically developing children (TDC). In children with cerebral palsy (CwCP), who have suffered an injury to the foetal or infant brain, acquisition of motor skills such as walking can be delayed or, in severe cases, completely missing (Sutherland, 1990). Currently, the gross motor functional classification system (GMFCS) is used to functionally categorize severity of deficits in motor function into five general levels (Rosenbaum et al., 2002). These categorization levels indicate the challenges faced by CwCP in acquiring the skills needed to walk continuously. For each GMFCS level, motor growth curves have been identified based on gross motor function measurement (GMFM), which, similar to growth charts (focussed mainly on the anthropometric variables of height and weight), provide an indication of age-related development. Importantly, although motor growth curves attempt to characterize varying degrees of deficit severity during maturation, its low-resolution limits discretisation of motor performance in pathological subjects, but it also does not allow to connect to the spectrum of healthy development. Consequently, motor growth curves cannot differentiate TDC from children with mild motor developmental delays (e.g., GMFCS I and II). Additionally, in high functioning CwCP, ceiling effects critically limit the child’s outlook since GMFM-score is not able to further improve after the age of 7–8 years within the motor growth curves (Hanna et al., 2008), despite clear evidence demonstrating progressive improvements are still possible in GMFCS I and II (Clutterbuck et al., 2021). In order to record/chart any further functional progress in a child we would need tools such as gait analysis coupled with our representation, over and above the GMFM. A high-resolution approach that allows TDC to be clearly indexed against pathological development, even in extremely mild cases such as GMFCS I, would clearly allow expectations of maturation and functional discrepancies to be managed effectively with parents, but also better designate underlying deficits and support clinical decision-making for targeted treatment programs.

In contrast to motor growth curves, functional movement patterns, including, e.g., fluctuations within a gait cycle, are known to continuously develop and change with age and skill acquisition (Bisi and Stagni, 2016; Gouelle et al., 2016; Caballero et al., 2019; Abram et al., 2022). During continuous locomotion, step repetitions are never constant, but rather fluctuate around a desired target. These fluctuations during walking, commonly referred to as gait variability, have shown promise in delineating complex neuromuscular traits such as skill exploration, adaptation, and development, but also impairment and degeneration (Hausdorff et al., 1985; Bisi and Stagni, 2016; Gouelle et al., 2016; Konig et al., 2016; Caballero et al., 2019; Ravi et al., 2020; Abram et al., 2022). When children learn to walk, they show high variability in their gait pattern, which then slowly decreases and remains relatively constant during adult life (Hausdorff et al., 1985; Bisi and Stagni, 2016; Gouelle et al., 2016; Sangeux et al., 2016). Further scoring methods using such parameters of gait variability such as the gait variability index (GVI, Gouelle et al., 2013), Pediatric Temporal–spatial Deviation Index (Zhou et al., 2019), and gaitSD (Sangeux et al., 2016), have thus been developed to provide a quantification of motor performance across different stages of maturation. However, since these assessment scores “bundle” different parameters and describe overall differences, they are not able to capture population heterogeneity in either degree or type of functional adaptations generally present in CwCP. One alternative to scoring approaches is to characterize differences in specific gait domains such as rhythm, asymmetry, and postural control (Lord et al., 2013), which are all inherently linked to the subject’s variability during gait. However, until now it remains unknown whether multiple domains of gait variability are sensitive to abnormalities in motor maturation, and hence able to discretise between healthy and GMFCS I/II. Thus, in this study, our aim was to investigate whether multiple domains of gait variability change across adolescence and with different levels of motor impairment.

By benchmarking the adaptation in gait variability and asymmetry that occurs from childhood to adulthood between GMFCS I/II and typically developing children, this study provides a functional map of (a)symptomatic motor development. Based on previous work (Hausdorff et al., 1985; Gouelle et al., 2016), our assumption was that development trajectories of gait variability follow a power law and we thus hypothesized that these trajectories will: 1) differ between CwCP and TDC, and 2) differ between mild and more severe forms of CP.

## 2. Materials and methods

### 2.1. Participants

For this cross-sectional retrospective study, 3D kinematics and clinical assessment data of CwCP or TDC were retrospectively extracted from the database of a local hospital. Informed consent was obtained from all children or their guardians, as approved by the local ethical committee (KEK Nr. 2018–01640). All measurements were conducted according to the Declaration of Helsinki. Participants were included if they had undergone 3DGA barefoot during regular clinical visits between August 2016 and May 2020, were aged 4–20 years at time of measurement, typically developing or diagnosed with spastic CP GMFCS levels I or II. Participants were excluded if they received Botulinum toxin injections within 6 weeks before the measurement, underwent surgical procedures within 12 months prior to the measurement, or did not have sufficient, at least 12 strides, 3DGA data available. The TDC were recruited through local schools and organizations and had participated in previous studies at the local hospital.

**Figure 1:**
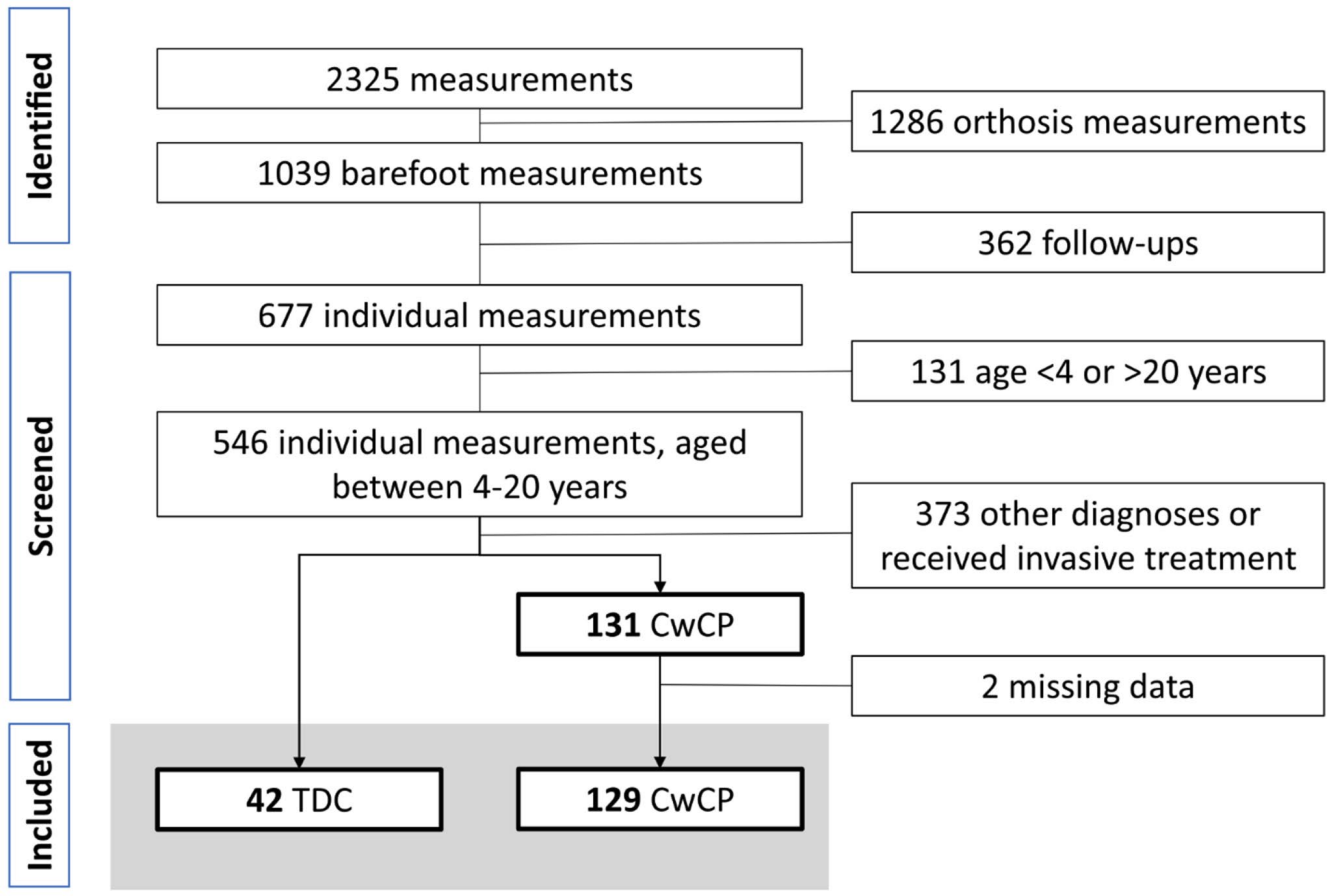
Flowchart participant selection. TDC, typically developing children and adolescents, CwCP, children and adolescents with cerebral palsy.

Within the recruitment period, 2,325 measurements were undertaken, of which 1,039 trials were conducted barefoot by 677 individuals. From these, 129 CwCP with GMFCS level I or II and 42 TDC were included (Figure 1).

### 2.2. Measurement procedure

All participants underwent clinical assessment (data on anthropometry, and in cases of CwCP the GMFCS level was assigned) before they walked barefoot on an instrumented walkway at their preferred walking speed. Each participant performed at least 5 gait trials over a 12 m instrumented walkway. Kinematic data was collected at a sampling frequency of 150 Hz using an optoelectronic motion capture system (12-cameras, MTX20, VICON, Oxford, United Kingdom). A total of 64 markers were attached to the subjects according to the Conventional Gait Model (CGM, Vicon, United Kingdom, 9.5 mm diameter markers) (Visscher et al., 2021).

### 2.3. Data processing and analysis

Pre-processing of the data (performed in VICON-NEXUS software package v2.8.2) included filtering of the data using the Woltring filter (mean squared error set to 10mm^2^, Woltring, 1986) Further analyzes were performed in MATLAB (The Mathworks Inc., vR2019a, Natick, United States) with the open-source Biomechanical ToolKit package (Barre and Armand, 2014). Trials considered to exhibit excessive soft tissue artifact, poor consistency, or signs of inaccurate marker placement were excluded by onsite gait analysis experts. Gait events (initial contact and toe-off) were identified by automatic detection using the sagittal velocity approach (Visscher et al., 2021). These gait events were then used to calculate spatio-temporal parameters (stride time – ST, stride length – SL, walking speed – WS, single support time – SS, cadence – CD, stride rather than step time/length were chosen as the data comprised unilaterally and bilaterally affected CwCP) for which the variability was expressed using the coefficient of variation (CV), where higher values indicate greater stride-to-stride variability (Table 1). Gait events were also used to normalize the temporal axis of the kinematic curves from the CGM to 100% of the gait cycle. From the 100% curves, the sagittal joint angles hip flexion, knee flexion, and ankle dorsiflexion (measured in °) were extracted, and their stride-to-stride fluctuations were expressed using the mean of the standard deviation (meanSD), where a higher meanSD indicated more variability. For both CV and meanSD, the values were first computed separately within each limb and then combined to avoid the potentially confounding effect of limb asymmetry (Lord et al., 2011). Asymmetry of gait was evaluated for SS (Asym-SS), ST (Asym-ST), and SL (Asym-SL) as the respective parameter ratio for each stride (Yogev et al., 2007), where zero indicated perfect symmetry between the limbs. The initial and the final captured strides were excluded from analysis, as well as strides with spatio-temporal gait parameters that were more than 3SD away from their mean, as our interest lay with quantifying steady-state walking.

**Table 1:** Overview of gait parameters, their definition, and how they were calculated.

	Parameter		Definition	Formula
Spatio-temporal variability	ST_CV	Stride time coefficient of variation	CV of time between two consecutive initial contacts on the same side	$CV=\left[\frac{SD}{\bar{x}}\right]\times 100\%$
	SL_CV	Stride length coefficient of variation	CV of distance traveled between two consecutive initial contacts on the same side	
	SS_CV	Single support coefficient of variation	CV of time when only one foot was in contact with the ground	
	WS_CV	Walking speed coefficient of variation	CV of distance covered by the whole body in a given time	
	CD_CV	Cadence coefficient of variation	CV of number of strides in a given time	
Asymmetry	Asym_ST	Stride time asymmetry	Ratio between shorter and longer stride time for each consecutive pair of strides	Asymmetry = $\ln\left|\frac{Short}{Long}\right|\times 100\%$
	Asym_SL	Stride length asymmetry	Ratio between shorter and longer stride length for each consecutive pair of strides	
	Asym_SS	Single support time asymmetry	Ratio between shorter and longer single support time for each consecutive pair of strides	
Kinematic variability	HipFlex	MeanSD of hip flexion angle	MeanSD of the rotation of the hip in the sagittal plane, normalized to the gait cycle	$MeanSD\;\left(\Delta x\right)=SD_{i}\left[x\right]$ fori∈{0%,…,100%}
	KneeFlex	MeanSD of Knee flexion angle	MeanSD of the rotation of the knee in the sagittal plane, normalized to the gait cycle	
	AnkleFlex	MeanSD of Ankle dorsiflexion angle	MeanSD of the rotation of the ankle in the sagittal plane, normalized to the gait cycle	

### 2.4. Statistical analysis

The assumption was made that gait variability follows a power law during maturation (Hausdorff et al., 1985; Gouelle et al., 2016), therefore a log10-transformation was applied before creating linear models in R (RStudio, R4.2.0, with libraries nlme, sfsmisc, ffplot2). Linear models were created for each gait parameters for asymmetry, spatio-temporal- or kinematic variability and consisted of a mixture of continuous (age) and factorial (group: CP or TD, GMFCS I or GMFCS II) parameters as shown here:

log(gait parameter)=log(Age)+Group+log(Age):group).

Output of the linear models were visually checked for normality and homoscedasticity of data distribution using the sfsmisc package from CRAN repository.^1^ A two-way analysis of variance (ANOVA) was applied to the outcomes of the linear models to test whether significant interaction effects existed between age and group for each of the selected parameters. Significance level was set at *α* = 0.05. Adjusted R-squared values were reported to indicate how well the model explained the data variation. Adjusted R-squared values between 0.02–0.13 were seen as weak, 0.14–0.26 as moderate, and > 0.26 as substantial (Cohen, 1988). To provide reference values for (a)symptomatic motor development from childhood to young adults, participants were divided into 3 age groups, 6–10, 11–15, and 16–20 years, that were defined prior to analysis. For each of these age categories, mean ± SD and 95% confidence intervals (95%CI, based in t-distribution to adjust for difference in sample size) based on the average values from each participant were used to benchmark the thresholds of upper and lower limits of variability between health status (TD and CP) and disease severity (GMFCS level I and II).

## 3. Results

Of the included 129 CwCP, 99 and 30 were classified as GMFCS I and II, respectively, (Table 2). For all groups, TDC and CwCP, all parameters of spatio-temporal and kinematic variability, as well as gait asymmetry, exhibited a negative correlation with the age groups (r between −0.6 and − 0.8, Supplementary material S2). Except for Asym_SL (*p* = 0.07), all parameters reduced significantly with age in the linear models (*p* < 0.05, Supplementary material S3).

**Table 2:** Participant description.

	CwCP		TDC
	GMFCS I	GMFCS II	
*N*	99	30	42^a^
Strides per participant (*n*)	37 (13) [12–83]	39 (14) [12–74]	27 (10) [12–61]
Age (years and months)	12y 4 m (3y.8 m) [6y 7 m -19y 11 m]	12y 6 m (3y.6 m) [7y 5 m – 18y 4 m]	12y 4 m (3y.8 m) [5y 6 m – 18y 8 m]
Gender (n_male_/n_female_)	66 / 33	18 / 12	21 / 21
Height (m)	1.49 (0.19) [1.11–1.86]	1.44 (0.16) [1.18–1.73]	1.47 (0.17) [1.20–1.77]
Weight (kg)	42.4 (17.2) [17.0–83.7]	39.8 (15.6) [17.6–81.3]	37.3 (12.0) [23.8–63.2]

Data is displayed as mean (SD) [range].

a Joint angles were only available for 20 out of 42 TDC, as a different marker model was used. CwCP, children and adolescents with cerebral palsy; TDC, typically developing children and adolescents; GMFCS, gross motor function classification system.

### 3.1. Typical & atypical development

**Figure 2:**
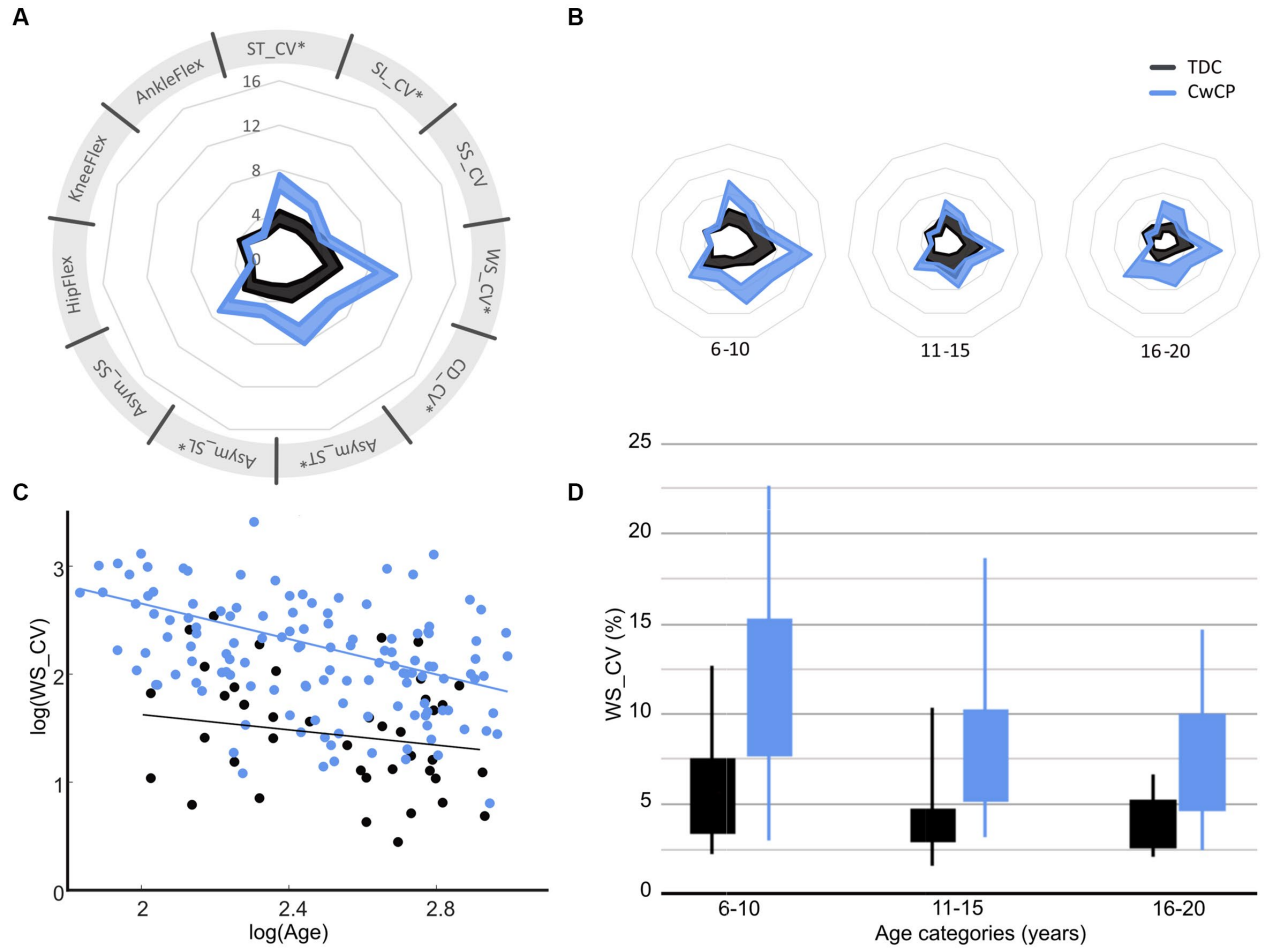
Confidence intervals (95%CI) of gait variability and asymmetry parameters for typical (TDC) and atypical (CwCP) development over all ages **(A)** and per age group **(B)**, with examples of how variability of walking speed reduces between age groups **(C,D)**. *Significant (*p* < 0.05) interaction effect with age. ST, stride time; SL, stride length; WS, walking speed; SS, single limb support time; CV, Cadence; Asym. Asymmetry; CV, coefficient of variation; HipFlex, meanSD of hip flexion angle; KneeFlex, meanSD of knee flexion angle; AnkleFlex, meanSD of ankle dorsiflexion angle. TDC, typically developing children and adolescents; CwCP, children and adolescents with cerebral palsy. Corresponding data for other gait parameters can be found in Supplementary material S1. Spyder-plot with SD instead of 95% CI can be found in Supplementary material S6.

A significant difference between TDC and CwCP was found for all parameters (*p* < 0.05, Supplementary material S1, Figure 2A), except the variability of joint angles (*p* = 0.29–0.87). The relation between the age groups and variability was significantly different between TDC and CwCP for some but not all parameters (*p* < 0.05 for ST_CV, SL_CV, WS_CV, CD_CV, Asym_ST, Asym_SL, Figure 2A, Supplementary material S1). Adjusted R-squared values from the linear models showed weak (SS_CV: 0.13) to moderate (Asym_SS: 0.17, Asym_ST: 0.18, SL_CV: 0.21, Asym_ST: 0.24, ST_CV: 0.26) to substantial (CD_CV: 0.28, WS_CV: 0.32, AnkleFlex: 0.32, KneeFlex: 0.35, HipFlex: 0.35, Figures 2C,D) goodness of fit.

### 3.2. GMFCS I & II

**Figure 3:**
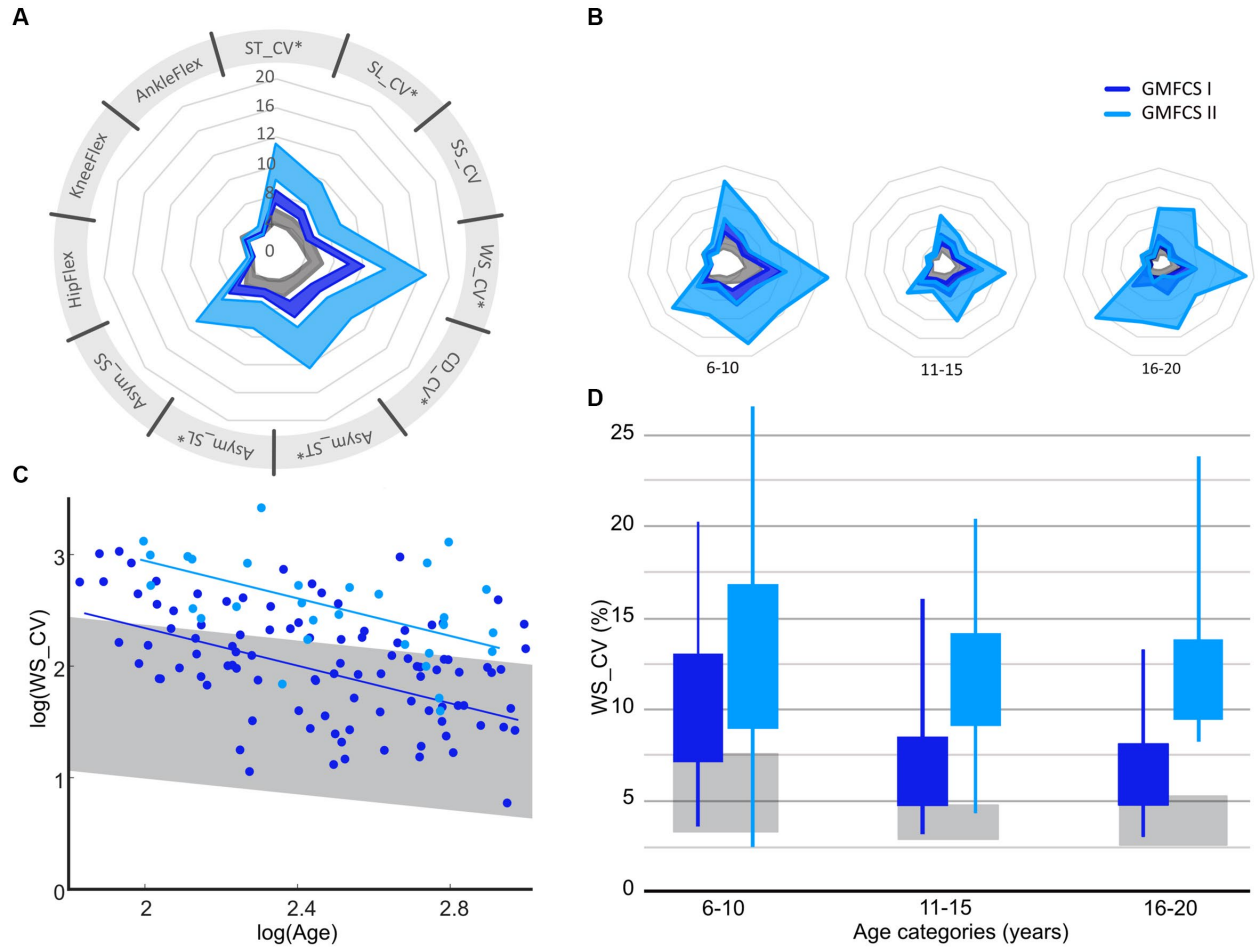
Confidence intervals (95% CI) of gait variability and asymmetry parameters for GMFCS I and GMFCS II over all ages **(A)** and per age group **(B)**, with examples of how variability of walking speed reduces between age groups **(C,D)**. GMFCS, gross motor function classification system; ST, stride time; SL, stride length; WS, walking speed; SS, single limb support time; CV, Cadence; Asym, Asymmetry; CV, coefficient of variation; HipFlex, meanSD of hip-flexion angle; KneeFlex, meanSD of knee-flexion angle; AnkleFlex, meanSD of ankle-dorsiflexion angle. Corresponding data for other gait parameters can be found in Supplementary material S2. Spyder-plot with SD instead of 95%CI can be found in Supplementary material S6.

A significant difference between the severity levels of CP was found for almost all parameters (*p* < 0.05, Supplementary material S2, Figure 3A), except for the kinematic variability of the knee flexion (*p* = 0.24). However, the change in parameters between the age groups was similar in both GMFCS levels (interaction *p* > 0.05 for all parameters, Figure 3, Supplementary material S2). Adjusted R-squared values showed moderate (Asym_SS: 0.16, Asym_ST: 0.18, Asym_ST: 0.20, SL_CV: 0.25, ST_CV: 0.28) to substantial (SS_CV: 0.31, CD_CV: 0.31, WS_CV: 0.33, meanSD_Knee: 0.34, meanSD_Ankle: 0.36, meanSD_Hip: 0.38) goodness of fit.

## 4. Discussion

To support clinical decision making for targeted treatment programs and effectively manage expectations from patients and their guardians, it is important to map gait maturation and functional discrepancies between TDC and CwCP. As the low-resolution of current tools limits their discretisation of motor performance and does not allow connection to the spectrum of healthy development, there is a clear requirement for a biomarker that fills this unmet need. Therefore, this study aimed to investigate whether multiple domains of gait – a clear multidomain expression of holistic neuromotor control status – change across adolescence and with different levels of motor impairment. In so doing, this study has benchmarked the adaptation in gait variability and asymmetry that occurs across childhood, adolescents, and adulthood between GMFCS I/II and typically developing children. Our findings showed that gait variability and asymmetry reduce between age-groups in children and adolescents with typical (TDC) and atypical (CwCP) development, but matured differently across age groups between TDC and CwCP in the temporo-spatial domain (Figure 2). Kinematic (joint angle) variability, on the other hand, showed a similar development in both TDC and CwCP. Between GMFCS I and GMFCS II, only a significant difference in the level of spatio-temporal variability and gait asymmetry was observed, these levels did not change (no significant interaction effect) across different age groups (Figure 3).

The decrease in gait variability between childhood and adolescence observed in our study is consistent with previous investigations (Hausdorff et al., 1985; Muller et al., 2013; Gouelle et al., 2016). The higher motor variability observed in younger children could allow purposeful exploration of motor space, when coupled with reinforcement, and can drive motor learning. While some reports state gait maturation is ongoing until the age of 8 years (Sutherland, 1997), our work further endorses the hypothesis that consistency of gait increases beyond this age group until skeletal maturity is reached (Hausdorff et al., 1985; Muller et al., 2013; Gouelle et al., 2016; Sangeux et al., 2016). The reduction in gait variability with age might follow the expected maturation of the neurophysiological system, which is known to continue into adulthood (Bethlehem et al., 2022), but further evaluation would be required to confirm this hypothesis. In CwCP, the reduction of spatio-temporal variability and gait asymmetry between age groups had a sharper decline than their typically developing peers, leading to less profound differences at older ages. When interpreting these results, it should be acknowledged that our cohort of CwCP received the standard of care in Switzerland (Supplementary material S5), which aims to normalize gait patterns towards those of TDC. Therefore, the observed reduction in gait variability and asymmetry between age groups of CwCP might be a result of the treatment rather than a natural progression of CP, but further research towards the effect of treatment on gait variability and asymmetry would be required to test this hypothesis. Due to the high diversity in philosophies for CP treatment around the world, it might not be possible to extrapolate the current findings to other countries. In future investigations, it would be advisable to include more data samples, especially in the younger and older age groups of TDC. Despite these limitations, the ability of our presented methods to discretise motor characteristics between TDC, GMFCS I, and GMFCS II highlights the potential for using developmental trajectories of gait variability and asymmetry as functional movement-based biomarkers – hence providing clear benefits for identifying children with mild movement related deficits, monitoring treatment responses, tracking possible deficit progression, and supporting the management of expectations.

Children and adolescents with GMFCS level II showed higher gait variability and asymmetry compared to those with GMFCS level I. However, the change between age groups was similar for both levels, indicating a similar amount of skill acquisition. It has to be acknowledged that CwCP with learning difficulties were mostly missing from our analyzes as participants who received recent treatment were excluded from participation in this study. On the other hand, highly functional individuals with extremely mild CP are likely missing from our dataset, since all our study participants were seen for clinical reasons (e.g., worsening of gait patterns). While these biases might limit the ecological validity of directly implementing our approach in clinical settings, our results clearly indicate that further development using larger prospective cohorts is warranted. What the current findings do show, however, is progressive decreases in gait variability in both GMFCS levels I and II from the youngest to the oldest age group. These continued improvements indicate that control of walking could still be enhanced across multiple domains of gait even though motor growth curves for the prognosis of gross motor function using GMFCS levels indicate a motor developmental plateau about the age of 7–8 years (Rosenbaum et al., 2002). Our presented developmental trajectories and spider plots can therefore complement the existing motor growth curves and further inform guardians, physicians, therapists, and other decision-makers about the improvement potential for motor performance in children and adolescents with mild forms of CP.

Gait variability and asymmetry are not only influenced by internal factors such as maturation and pathology, but external factors including environment and perturbations can also have an impact (Chau et al., 2005). This plausibly explains why the 95% CIs reported in Supplementary materials S1, S2 do not overlap with values previously reported (Hausdorff et al., 1985; Muller et al., 2013). When comparing values and methodologies from different studies, it becomes clear that longer walking durations are associated with lower CV values. Ideally 30–60 strides are used to assess spatio-temporal variability reliably in an individual (Konig et al., 2014; Sangeux et al., 2016). However, in standard clinical settings such numbers are not always available or possible. To assess the influence of the number of strides on our outcomes, we conducted a short examination by taking a sub-sample of the dataset where at least 30 strides per participant were available (Supplementary material S3). No meaningful differences in group averages were identified. We therefore consider it most important that conditions are similar between measurement settings. Another factor that could influence the inherent variability observed is a change in experimenters conducting the CGA session. As all the data within this study were collected within the same environment, by a small group of experts trained on, identical assessment of protocols, we deem the influence to be minor. No *a priori* sample size estimation could be performed as we investigated changes in gait variability with age and the relevant values comparing TDC and CwCP were not available within the literature. Adhering to our inclusion/exclusion criteria all possible datasets in the local hospital database were included for analysis. Our results can form the basis for sample size evaluations for future studies.

**Figure 4:**
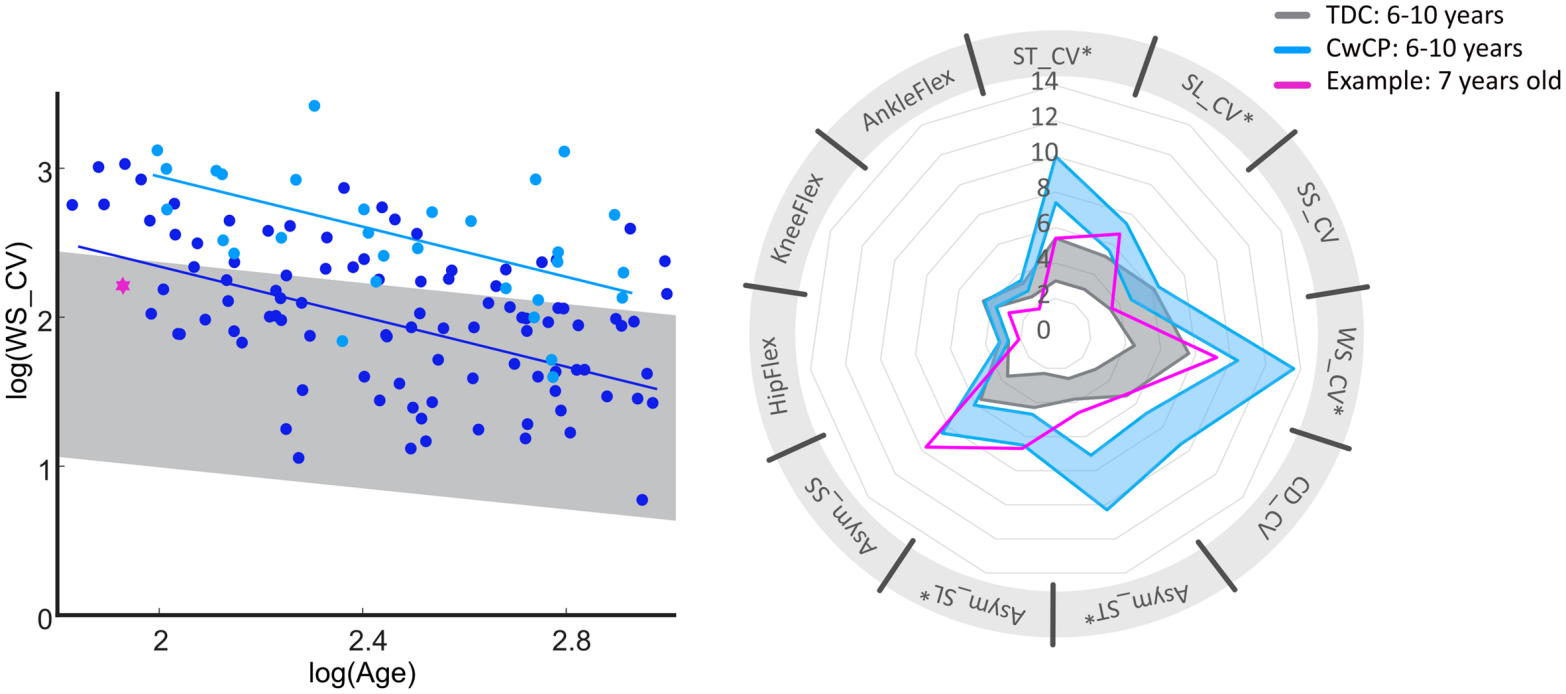
Overview of deficits in gait variability and asymmetry of one exemplary subject (pink) diagnosed with spastic unilateral CP (top). Reference values for GMFCS I (dark blue) and typical development (gray) for age group 6–10 years are provided within the graphs. Bottom of the figure provides general information on the exemplary subject (pink, dotted line represents the right limb), with reference to TDC (gray). GMFCS, gross motor function classification system; CwCP, children with cerebral palsy; TDC, typically developing children; ST, stride time; SL, stride length; WS, walking speed; SS, single limb support time; CV, Cadence; Asym, Asymmetry; CV, coefficient of variation; HipFlex, meanSD of hip-flexion angle; KneeFlex, meanSD of knee-flexion angle; AnkleFlex, meanSD of ankle-dorsiflexion angle.

The concept of developmental trajectories for gait variability and asymmetry presented in our study has the potential provide milestones on age-related changes for multiple gait domains. It is of interest to consider gait domains independently as these are thought to be governed by various neurophysiological structures, which mature at different ages (Grillner and El Manira, 2020; Bethlehem et al., 2022). Furthermore, development in gait variability will also follow growth in anthropometry (World Health Organization, 2023) as well as in motor control and patterns (Dominici et al., 2011), and development of walking (Thelen and Cooke, 1987). However, further research is needed to understand the inter-relationships between the developmental trajectories of gait variability and asymmetry and the maturation of structures responsible for neurophysiological and motor functions. Considering the difference in skeletal and neurophysiological maturation between males and females, separate curves for each sex would be advisable for further improvement of the developmental trajectories for gait variability and asymmetry. Additionally, a previous study reported no differences in gait variability between those suffering from uni- vs. bilateral CP (Tabard-Fougèrel et al., 2022). Instead, the authors reported differences between the affected and less-affected side. Therefore, it might of interest in the future to focus on the differences between the two sides within an individual and then compare those across other patient types. In addition to providing insight into each individual’s neurophysiological status, discretising deficits in gait parameters is of importance to allow domain-specific treatment options (Bakir et al., 2013). For example, one participant who was classified as GMFCS I at the age of 6.9 years, exhibited deficits in spatial variability and the gait domain “asymmetry.” For this individual, community activities that challenge balance and require precise movements might be useful, such as dancing (Figure 4) (Patterson et al., 2018; Liu et al., 2022). Importantly, however, the standard-of-care clinical classification of motor growth curve GMFCS level I was not able to identify these specific deficits or provide expectation management beyond the child’s current status. Future research could further investigate the effect of specific treatments or other meaningful activities on motor maturation. As early intervention is often considered key in optimizing outcomes, expanding the current datasets to include trajectories from early childhood could provide early prognostic clues in motor adaptation while also providing possible links to current research on motor control and walking development in toddlers (Bekius et al., 2021). Furthermore, with the advent of portable digital technologies it would also be possible to extend this cross-sectional research approach to also include longitudinal datasets, such that modifications can be mapped at the level of the individual.

Overall, this study has benchmarked the difference in gait variability and asymmetry that occurs across childhood, adolescents, and adulthood between high functioning CwCP and those with typical development by providing a first functional map of (a)symptomatic motor developmental trajectories. Such trajectories exhibit clear potential for use as functional biomarkers towards identifying mild (Pediatric) movement related deficits, monitoring treatment response, and supporting management of expectations.

## Data availability statement

The datasets presented in this study can be found in online repositories. The names of the repository/repositories and accession number(s) can be found at: https://www.research-collection.ethz.ch/handle/20.500.11850/582704.

## Ethics statement

The studies involving humans were approved by Cantonal Ethics Committee, Zurich, Switzerland (KEK 2018-01640). The studies were conducted in accordance with the local legislation and institutional requirements. Written informed consent for participation in this study was provided by the participants’ legal guardians/next of kin.

## Author contributions

RV, NS, and RB: conceptualisation. RV, MG, and RB: data curation. RV and NS: formal analysis and methodology. WT and RB: funding acquisition. RV, MG, and NS: investigation and writing – original draft. RV: project administration, validation, and visualization. EV, WT, and RB: resources. EV, WT, RB, and NS: supervision. RV, MG, EV, WT, RB, and NS: writing – review & editing. All authors contributed to the article and approved the submitted version.

## Funding

This study was financially supported by the Ralf-Loddenkemper Foundation under grant CH-270.7.002.704-3. Open access funding was provided by ETH Zurich.

## Conflict of interest

The authors declare that this research was conducted in the absence of any commercial or financial relationships that could be construed as a potential conflict of interest.

## Publisher’s note

All claims expressed in this article are solely those of the authors and do not necessarily represent those of their affiliated organizations, or those of the publisher, the editors and the reviewers. Any product that may be evaluated in this article, or claim that may be made by its manufacturer, is not guaranteed or endorsed by the publisher.

## Footnotes

^1^ https://rdrr.io/cran/sfsmisc/man/TA.plot.html
