# Supplementary material for: Can developmental trajectories in gait variability provide prognostic clues in motor adaptation among children with mild cerebral palsy? A retrospective observational cohort study
Source: Front Hum Neurosci. 2023 Sep 19;17:1205969. doi: 10.3389/fnhum.2023.1205969 (PMC10546019; doi:10.3389/fnhum.2023.1205969)

Supplementary materials

To Visscher et al. 2023 "Can developmental trajectories in gait variability provide prognostic clues in motor adaptation among children with mild cerebral palsy? A retrospective observational cohort study"

- **S1. Outcomes of the comparison between TDC and CwCP**
  - **Table 1. Outcomes of log-transferred linear models on gait variability and asymmetry between typical (TDC) and atypical (CwCP) development**
  - **Table 2. 95% confidence interval per age group for typical (TDC) and atypical (CwCP) development**
- **S2. Outcomes of the comparison between GMFCS I and II**
  - **Table 1. Outcomes of log-transferred linear models on gait variability and asymmetry between typical (TDC) and atypical (CwCP) development**
  - **Table 2. 95% confidence interval per age group for typical (TDC) and atypical (CwCP) development**
- **S3. Evaluation of the effect of the number of included strides on gait variability and asymmetry outcomes**
- **S4. Example of how the presented concept of motor-developmental trajectories for gait variability can be used in practice**
- **S5. Standard care of CP in Switzerland**
- **S6. Confidence interval (95%CI) vs. standard deviation (SD) of gait variability and asymmetry parameters over all ages**
  - **Figure 1. Comparison between typical (TDC) and atypical (CwCP) development**
  - **Figure 2. Comparison between GMFCS I and GMFCS II**

S1. Outcomes of the comparison between TDC and CwCP

S1 Table 1: Outcomes of log-transferred linear models on gait variability and asymmetry between typical (TDC) and atypical (CwCP) development. *$p<0.05$, **$p<0.001$, ***$p<0.0001$. TDC: typically developing children and adolescents, CwCP: children and adolescents with mild cerebral palsy, ST: stride time, SL: stride length, WS: walking speed, SS: single limb support time, CD: cadence, CV: coefficient of variation, Asym: Asymmetry, HipFlex: mean of the standard deviation (meanSD) of the hip-flexion angle, KneeFlex: meanSD of the knee-flexion angle, AnkleFlex: meanSD of the ankle-dorsiflexion angle.

	TDC		CwCP		ANOVA – *p* values			Pearson correlation	Adjusted-R^2
	mean±SD	CI-95%	mean±SD	CI-95%	age	group	interaction	age	
ST_CV (%)	3.7±2.1	3.0-4.3	6.8±4.1	6.1-7.6	***	***	0.02*	-0.7	0.26
SL_CV (%)	3.5±1.8	2.9-4.0	5.5±2.9	5.0-6.0	0.002 **	***	0.01*	-0.6	0.21
WS_CV (%)	5.0±2.7	4.2-5.6	9.7±5.0	8.8-10.6	***	***	0.01*	-0.7	0.32
SS_CV (%)	3.8±2.0	3.1-4.4	4.5±2.5	4.0-4.9	***	0.04*	0.11	-0.6	0.13
CD_CV (%)	3.5±1.9	2.9-4.0	6.2±3.7	5.6-6.9	***	***	0.02*	-0.8	0.28
Asym_ST (%)	3.3±2.4	2.5-4.0	7.1±5.2	6.2-8.0	0.005 **	***	0.013*	-0.7	0.24
Asym_SL (%)	3.0±1.8	2.4-3.5	7.9±2.8	4.4-5.4	0.07	***	0.037*	-0.4	0.21
Asym_SS (%)	3.6±1.8	3.0-4.2	6.5±3.9	5.8-7.2	0.019 *	***	0.76	-0.3	0.16
HipFlex (°)	2.6±0.5	2.4-2.9	2.4±0.8	2.3-2.6	***	0.29	0.89	-0.6	0.35
KneeFlex (°)	3.6±0.9	3.2-4.0	3.3±1.1	3.2-3.5	***	0.44	0.47	-0.6	0.35
AnkleFlex (°)	2.6±0.8	2.3-3.0	2.6±1.0	2.4-2.8	***	0.87	0.94	-0.7	0.32

S1 Table 2: Values per age group for typical (TDC) and atypical (CwCP) development. TDC: typically developing children and adolescents, CwCP: children and adolescents with mild cerebral palsy, ST: stride time, SL: stride limb length, WS: walking speed, SS: single support time, CD: cadence, CV: coefficient of variation, Asym: Asymmetry, HipFlex: mean of the standard deviation (meanSD) of the hip-flexion angle, KneeFlex: meanSD of the knee-flexion angle, AnkleFlex: meanSD of the ankle-dorsiflexion angle.

	6-10years				11-15 years				16-20 years			
	TDC N=17/12		CwCP N=50		TDC N=13/5		CwCP N=52		TDC N=12/3		CwCP N=27	
	mean±SD	CI-95%	mean±SD	CI-95%	mean±SD	CI-95%	mean±SD	CI-95%	mean±SD	CI-95%	mean±SD	CI-95%
**ST_CV (%)**	4.2±2.4	3.0-5.4	8.7±4.7	7.4-10.0	3.9±2.2	2.8-5.1	5.7±3.3	4.8-6.7	2.3±0.8	1.7-2.8	5.5±2.8	4.4-6.6
**SL_CV (%)**	4.1±2.1	3.0-5.2	6.5±3.2	5.6-7.4	3.1±1.8	2.2-4.1	4.8±2.1	4.2-5.3	3.0±1.1	2.0-3.8	5.0±3.1	3.7-6.2
**WS_CV (%)**	6.0±3.0	4.5-7.6	12.0±5.6	10.4-13.6	4.6±2.6	3.2-6.0	8.4±4.0	7.3-9.5	3.8±1.6	2.5-5.0	8.0±4.3	6.3-9.7
**SS_CV (%)**	4.7±2.6	3.4-6.1	5.6±2.9	4.7-6.4	3.2±1.4	2.4-3.9	3.8±1.7	3.3-4.3	3.0±0.9	2.3-3.7	3.7±1.9	3.0-4.4
**CD_CV (%)**	4.1±2.2	3.0-5.3	8.1±4.4	6.8-9.4	3.5±1.6	2.6-4.3	5.1±2.7	4.3-5.8	2.2±0.7	1.6-2.7	5.0±2.5	4.0-6.0
**Asym_ST (%)**	3.2±1.2	2.6-3.8	8.7±5.6	7.1-10.3	4.1±3.5	2.2-6.0	6.3±4.8	5.0-7.6	2.0±0.7	1.4-2.5	5.7±4.4	3.9-7.4
**Asym_SL (%)**	3.3±1.9	2.3-4.3	5.6±3.2	4.7-6.5	3.0±1.9	2.0-4.0	4.2±2.0	3.7-4.8	2.3±1.2	1.4-3.2	4.7±3.1	3.5-6.0
**Asym_SS (%)**	4.6±1.9	3.6-5.6	7.3±4.2	6.1-8.5	3.4±1.6	2.5-4.2	5.8±3.0	5.0-6.6	2.2±0.5	1.8-2.6	6.4±4.9	4.5-8.4
**HipFlex (°)**	2.9±0.4	2.7-3.2	3.0±0.7	2.7-3.2	2.2±0.3	1.9-2.6	2.2±0.6	2.1-2.4	2.1±0.2	1.6-2.5	1.9±0.6	1.7-2.2
**KneeFlex (°)**	4.1±0.6	3.7-4.5	4.1±1.1	3.7-4.4	2.9±0.6	2.2-3.6	3.0±0.7	2.8-3.2	2.6±0.4	1.6-3.6	2.7±0.8	2.4-3.1
**AnkleFlex (°)**	3.0±0.7	2.5-3.4	3.2±1.2	2.9-3.6	2.2±0.7	1.3-3.1	2.3±0.6	2.1-2.4	1.9±0.5	0.7-3.2	2.1±0.7	1.8-2.4

S2. Outcomes of the comparison between GMFCS I and GMFCS II

S2 Table 1: Outcomes of log-transferred linear models on gait variability and asymmetry between GMFCS levels I and II. *p<0.05, **p<0.001, ***p<0.0001. GMFCS: gross motor function classification system, ST: stride time, SL: stride length, WS: walking speed, SS: single limb support time, CD: cadence, CV: coefficient of variation, Asym: Asymmetry, HipFlex: mean of the standard deviation (meanSD) of the hip-flexion angle, KneeFlex: meanSD of the knee-flexion angle, AnkleFlex: meanSD of the ankle-dorsiflexion angle.

	GMFCS I		GMFCS II		ANOVA – *p* values			Pearon correlation	R^2adjusted
	mean±SD	CI-95%	mean±SD	CI-95%	age	group	interaction	age	
ST_CV (%)	6.0±3.3	5.3-6.7	9.7±5.0	7.8-11.6	***	***	0.26	-0.6	0.28
SL_CV (%)	4.9±2.6	4.4-5.4	7.4±3.7	6.1-8.8	***	***	0.47	-0.6	0.25
WS_CV (%)	8.5±4.1	7.7-9.3	13.7±5.8	11.5-15.8	***	***	1.00	-0.7	0.33
SS_CV (%)	3.9±1.9	3.5-4.3	6.2±3.2	5.0-7.4	***	***	0.22	-0.6	0.31
CD_CV (%)	5.5±3.1	4.9-6.1	8.7±4.5	7.1-10.4	***	***	0.52	-0.7	0.31
Asym_ST (%)	6.1±4.4	5.2-7.0	10.3±6.0	8.1-12.6	***	***	0.49	-0.7	0.21
Asym_SL (%)	4.3±2.1	3.9-4.7	6.7±3.9	5.3-8.2	0.004**	***	0.28	-0.5	0.18
Asym_SS (%)	5.7±3.3	5.1-6.4	9.1±4.8	7.4-10.9	0.02*	***	0.83	-0.4	0.16
HipFlex (°)	2.4±0.8	2.2-2.5	2.7±0.7	2.4-3.0	***	0.002**	0.35	-0.6	0.38
KneeFlex (°)	3.3±1.1	3.1-3.5	3.4±1.0	3.1-3.8	***	0.24	0.18	-0.7	0.34
AnkleFlex (°)	2.5±0.7	2.3-2.7	2.9±1.3	2.4-3.4	***	0.015*	0.74	-0.7	0.35

S2 Table 2: 95% confidence intervals per age group for GMFCS levels I and II. GMFCS: gross motor function classification system, ST: stride time, SL: stride length, WS: walking speed, SS: single limb support time, CD: cadence, CV: coefficient of variation, Asym: Asymmetry, HipFlex: mean of the standard deviation (meanSD) of the hip-flexion angle, KneeFlex: meanSD of the knee-flexion angle, AnkleFlex: meanSD of the ankle-dorsiflexion angle.

	6-10years				11-15 years				16-20 years			
	GMFCS I N=39		GMFCS II N=11		GMFCS I N=39		GMFCS II N=13		GMFCS I N=21		GMFCS II N=6	
	mean±SD	CI-95%	mean±SD	CI-95%	mean±SD	CI-95%	mean±SD	CI-95%	mean±SD	CI-95%	mean±SD	CI-95%
**ST_CV (%)**	7.5±3.7	6.3-8.7	12.9±5.7	9.1-16.8	5.1±3.0	4.1-6.0	7.7±3.6	5.5-9.9	4.8±2.3	3.8-5.9	7.9±3.4	4.4-11.5
**SL_CV (%)**	5.8±2.7	5.0-6.7	8.9±4.0	6.2-11.6	4.5±2.1	3.8-5.1	5.6±2.1	4.4-6.9	3.9±1.6	3.2-4.6	8.7±4.5	3.9-13.4
**WS_CV (%)**	10.6±4.4	9.1-12.0	17.1±6.5	12.8-21.5	7.5±3.6	6.4-8.7	11.1±4.0	8.6-13.5	6.5±2.8	5.3-7.8	13.0±5.0	7.7-18.3
**SS_CV (%)**	4.8±2.2	4.1-5.6	8.2±3.8	5.7-10.7	3.4±1.6	2.9-3.9	4.9±1.8	3.7-6.0	3.2±1.0	2.7-3.7	5.5±3.0	2.4-8.6
**CD_CV (%)**	7.1±3.7	5.9-8.3	11.5±5.3	8.0-15.1	4.4±2.2	3.7-5.1	7.1±3.2	5.1-9.0	4.4±2.0	3.4-5.3	7.2±2.7	4.4-10.1
**Asym_ST (%)**	7.5±4.7	5.9-9.0	13.0±6.5	8.7-17.3	5.5±4.2	4.1-6.8	8.9±5.5	5.6-12.2	4.9±3.8	3.1-6.6	8.5±5.6	2.7-14.4
**Asym_SL (%)**	5.0±2.1	4.3-5.7	7.9±4.9	4.6-11.2	4.0±2.1	3.3-4.7	4.9±1.6	3.9-5.9	3.7±1.7	2.9-4.5	8.4±4.1	4.1-12.7
**Asym_SS (%)**	6.3±3.4	5.2-7.4	10.8±4.9	7.4-14.1	5.3±2.8	4.4-6.2	7.4±2.9	5.6-9.1	5.4±3.7	3.7-7.1	10.0±7.0	2.6-17.3
**HipFlex (°)**	2.9±0.7	2.6-3.1	3.2±0.8	2.7-3.8	2.2±0.6	2.0-2.4	2.4±0.5	2.1-2.7	1.8±0.6	1.5-2.0	2.5±0.5	2.0-3.1
**KneeFlex (°)**	4.0±1.1	3.7-4.4	4.1±1.1	3.4-4.8	2.9±0.7	2.7-3.2	2.9±0.7	2.5-3.3	2.6±0.8	2.2-2.9	3.4±0.6	2.7-4.0
**AnkleFlex (°)**	3.1±1.0	2.8-3.4	3.7±1.7	2.6-4.8	2.2±0.7	2.0-2.4	2.4±0.6	2.0-2.7	2.0±0.7	1.6-2.3	2.6±0.8	1.8-3.3

S3. Evaluation of the effect of the number of included strides on gait variability and asymmetry outcomes

As the number of strides used within the calculation for gait variability is known to influence the outcomes, we tried to quantify this effect to ensure it did not affect our results. From our participants, we selected the individuals from whom at least 30 strides were available. At random we included 2/4/6/8/10/12 left strides and 2/4/6/8/10/12 right strides to calculate everyone's gait variability (grey and blue representing respectively children and adolescents with TD or CP) and the group average (black). This procedure was repeated a hundred times. The findings were visualized using boxplots. While the variation on individual level was large, the group averages showed little variation. TD: typically developing, CP: cerebral palsy.

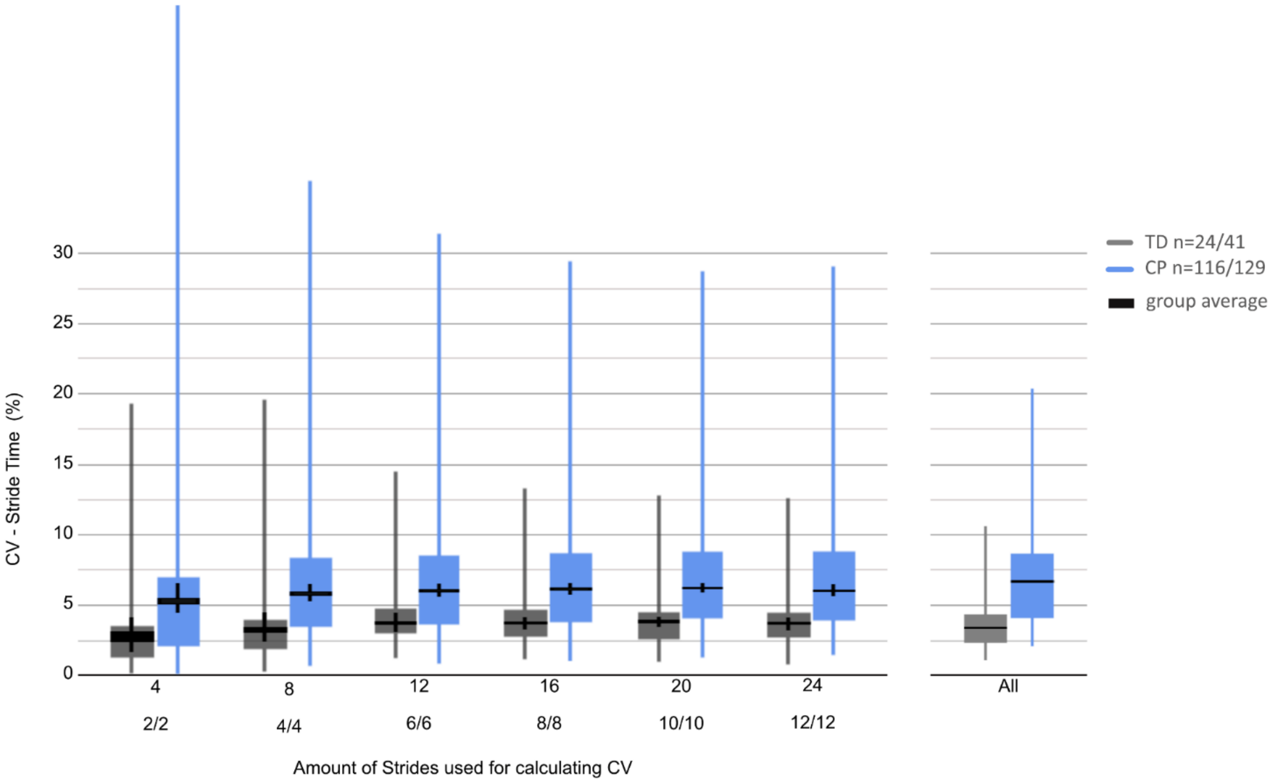

S4. Example of how the presented concept of motor-developmental trajectories for gait variability can be used in practice

The spider plot can support identification of motor deficits in specific gait domains while the scatter plot can help visualize and track changes over time. In pink the values and lines are shown of an example subject 6 years and 11 months old, in green you see the same values of the participant 1 year and 6 months later, after receiving physiotherapy 2x a week.

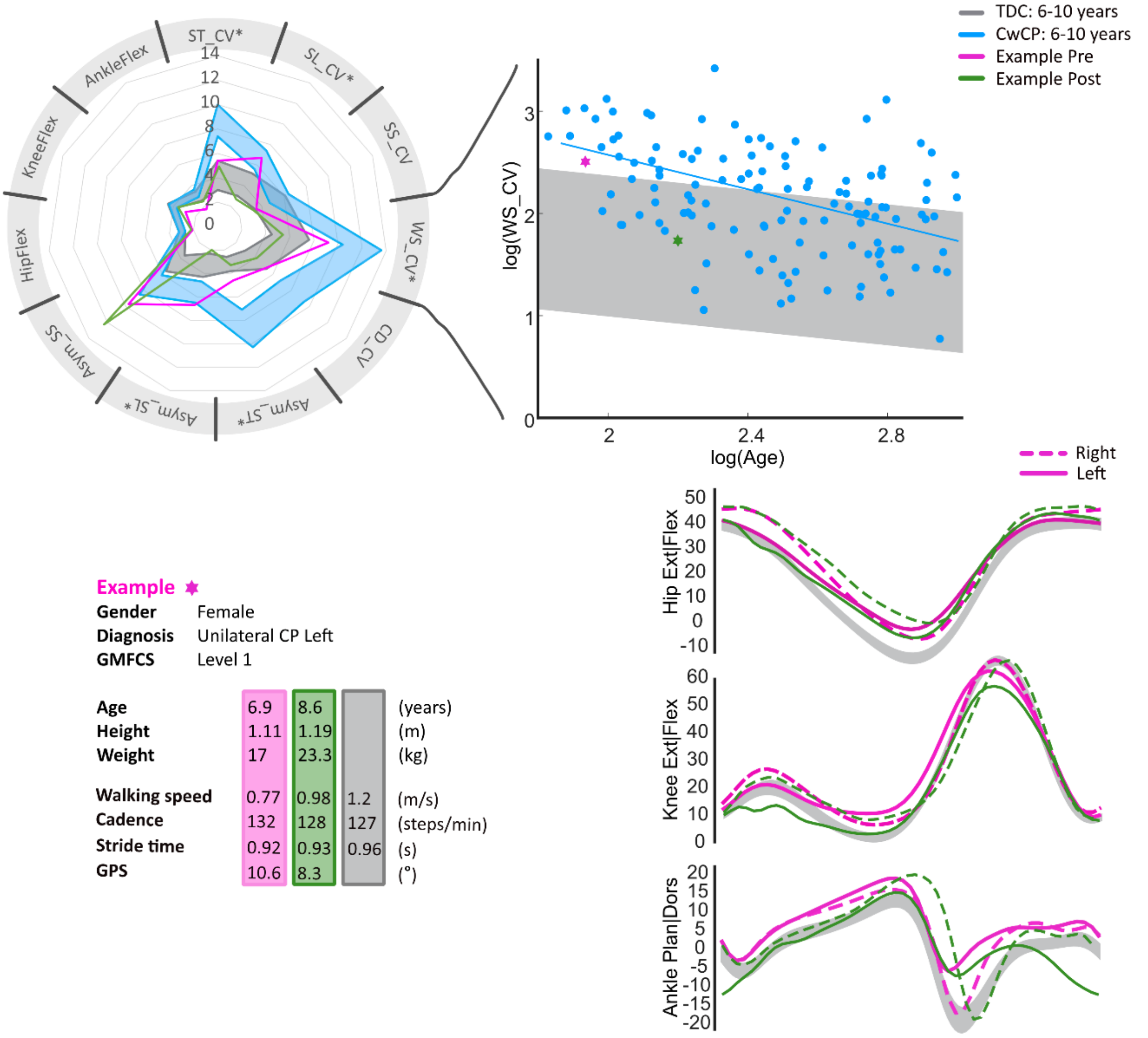

S5. Standard care of CP in Switzerland

Each newborn is scored for risk factors. Most of the CP cases are detected this way. Few more are detected later during routine screening exams by the pediatrician. When diagnosed, a neuropediatrician is involved as soon as possible, as soon as upright activities (sitting, standing, walking) are initiated an orthopaedic surgeon with special knowledge in neuroorthopaedics is additionally added to the care team. More or less all patients get physiotherapy (45 mins per session once to twice a week) on a regular basis. The physiotherapy content involves motor development, muscle strengthening, and maintenance of muscle length and joint mobility. In case of severe deficits in equilibrium, hippotherapy and occupational therapy are also prescibed, the latter also for problems of the upper extremity. Functional orthotics are applied as soon as required with the aim of controlling and preventing foot deformities. These functional orthotics are considered to provide stability and functional improvement. For this reason, more functional and much less positional orthotics are used. Botulinum toxin is administered cautiously and only occasionally. From age 6- 7 years onwards, gait analysis is performed to better understand the functional disorder and the possibilities of treatment. Surgery is avoided until the start of puberty (if possible). X-rays are carried out for hip and spine controls as indicated apart from a locally based hip screening program for GMFCS III-V.

S6. Standard deviation (SD) of gait variability and asymmetry parameters over all ages

Figure 1. Comparison between typical (TDC) and atypical (CwCP) development

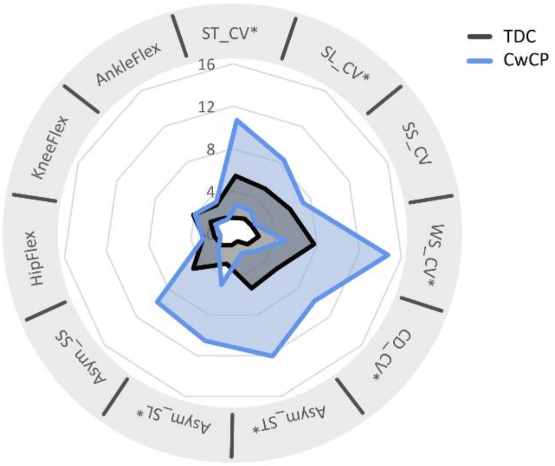

Figure 2. Comparison between GMFCS I and GMFCS II

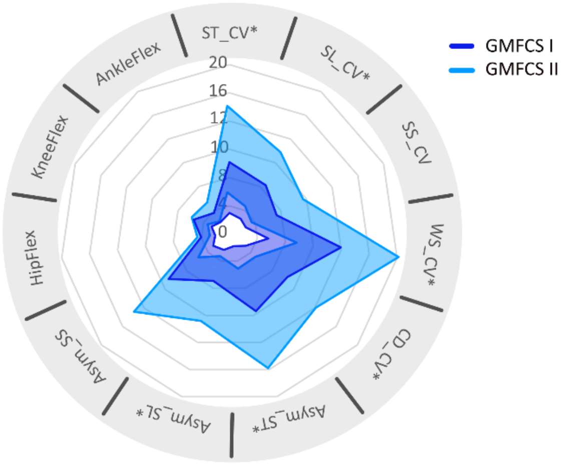

Supplement: Supplementary file 1 [file Data_Sheet_1.PDF]
